# Editorial: Diabetes complications: navigating challenges and unveiling new solutions

**DOI:** 10.3389/fendo.2026.1858882

**Published:** 2026-04-29

**Authors:** Khalid Siddiqui, Thorsten Siegmund

**Affiliations:** 1 Department of Biochemistry, College of Medicine, Kuwait University, Kuwait, Kuwait; 2 Division of Endocrinology and Diabetology, Isar Clinic, Munich, Germany

**Keywords:** cardiovascular and metabolic complications, diabetes complications, diabetic foot complications, diabetic kidney disease, diabetic retinopathy, neuropathy and musculoskeletal complications

Diabetes mellitus remains a major global health concern, affecting 589 million adults worldwide and continuing to rise across all regions (1). Although improvements in glucose management have reduced some risks, diabetes-related complications still cause substantial morbidity, mortality, and healthcare costs (2). These complications include both microvascular and macrovascular conditions, such as nephropathy, retinopathy, neuropathy, cardiovascular disease, and peripheral arterial disease. In addition, emerging evidence highlights other important outcomes, including cognitive impairment and sarcopenia, which further increase the burden of disease (3, 4). The Research Topic “Diabetes Complications: Navigating Challenges and Unveiling New Solutions” brings together studies that examine the complexity of diabetic complications and the need for earlier detection, better risk prediction, and more personalized treatment strategies. By integrating advances in clinical research and translational science, this Research Topic aims to improve understanding of the mechanisms underlying diabetic complications and support more effective prevention and management approaches. Taken together, these studies contribute to the broader effort to reduce the long-term impact of diabetes and improve patient outcomes worldwide.

## Cardiovascular and metabolic complications: novel risk markers

Cardiovascular disease remains the leading cause of mortality in individuals with type 2 diabetes, necessitating improved risk stratification tools. Several studies in this Research Topic highlight the emerging role of the triglyceride-glucose (TyG) index as a valuable biomarker for metabolic complications. Sun et al. demonstrated a significant association between the TyG index and hyperuricemia in patients with type 2 diabetes, suggesting that this simple, cost-effective marker could identify individuals at elevated cardiovascular risk. Complementing this finding, Chen et al. revealed a dose-response relationship between the TyG index and metabolic syndrome risk in a cross-sectional analysis of 1,560 patients, underscoring the index’s potential utility in clinical practice.

The intersection of lipid metabolism and atherosclerotic cardiovascular disease (ASCVD) in the context of diabetic kidney disease (DKD) was explored by Li et al., who identified a nonlinear relationship that transforms into a linear association, providing new insights into the complex interplay between cardiovascular and renal complications. Furthermore, Men et al. investigated the atherogenic index of plasma in relation to inflammatory markers and DKD, highlighting the inflammatory component of diabetic complications and the need for integrated management approaches.

The real-world adherence to organ-protective therapies was examined by Suliman et al., who found significant gaps in the implementation of guideline-recommended treatments for patients with type 2 diabetes and concurrent cardiovascular disease, heart failure, or kidney disease. This study emphasizes the critical need for healthcare system improvements and enhanced patient education to optimize therapeutic outcomes.

## Diabetic kidney disease: from risk stratification to precision management

Diabetic kidney disease represents a major complication affecting approximately 40% of individuals with diabetes. The studies in this Research Topic advance our understanding of DKD through multiple lenses. Meng et al. proposed a groundbreaking multimodal framework that integrates digital phenotypes and clinical biomarkers for risk stratification and precision management of diabetic kidney disease, representing a paradigm shift toward personalized medicine.

The epidemiological landscape of diabetic nephropathy in China was comprehensively analyzed by Zhang et al., who examined trends, age-period-cohort effects, and projections of disease burden from 1990 to 2036 using Global Burden of Disease data. This large-scale analysis provides crucial insights into public health planning and resource allocation. The association between diabetic retinopathy and diabetic kidney disease was further explored by Wu et al. in a prospective observational study, reinforcing the interconnected nature of microvascular complications and the importance of comprehensive screening protocols.

## Diabetic retinopathy: therapeutic innovations and mechanistic insights

Diabetic retinopathy remains the leading cause of preventable blindness in working-age adults. This Research Topic includes several important contributions to understanding and managing this complication. Zhang et al. investigated the anti-inflammatory effects of dexamethasone implants in eyes with heavy silicone oil following vitrectomy for diabetic retinopathy, demonstrating promising therapeutic benefits. At the molecular level, El-Asrar et al. elucidated the key role of the PGC-1α/ERR-α pathway in regulating angiogenic factors in proliferative diabetic retinopathy, opening new avenues for targeted therapeutic interventions.

Interestingly, Yang and Wu employed Mendelian randomization to evaluate the efficacy and safety of acupuncture for diabetic retinopathy, representing an innovative approach to assessing complementary therapies using genetic epidemiology methods. This study exemplifies the integration of traditional medicine with modern research methodologies.

## Neuropathy and musculoskeletal complications: emerging concerns

Diabetic neuropathy affects up to 50% of individuals with long-standing diabetes, yet early detection and prediction remain challenging. Sun et al. developed and validated a machine learning-based prediction model for diabetic peripheral neuropathy, demonstrating the potential of artificial intelligence in enhancing clinical decision-making and enabling earlier intervention.

An emerging area of concern is diabetic sarcopenia, characterized by loss of muscle mass and function in individuals with diabetes. Yin et al. conducted a comprehensive scoping review of screening tools for diabetic sarcopenia in type 2 diabetes mellitus, highlighting the need for standardized assessment methods and increased clinical awareness of this underrecognized complication.

## Diabetic foot complications: infection management and risk prediction

Diabetic foot complications, including ulcers and osteomyelitis, represent a major cause of hospitalization and lower-limb amputation. Liu et al. performed a systematic review and meta-analysis to determine the diagnostic value and integrated threshold of erythrocyte sedimentation rate (ESR) for diabetic foot osteomyelitis, providing evidence-based guidance for clinicians. Additionally, Zang et al. developed and validated a risk prediction model for multidrug-resistant organisms infection in diabetic foot ulcer patients, addressing a critical clinical challenge in an era of increasing antimicrobial resistance. The analysis by Pu et al. further characterized pathogen distribution and serum procalcitonin level alterations in patients with type 2 diabetes complicated by urinary tract infections, emphasizing the importance of tailored antimicrobial strategies.

## Cognitive impairment and hepatic complications: expanding the complication spectrum

The recognition that diabetes affects multiple organ systems beyond the traditional complications continues to expand. Zhao et al. conducted a systematic review and meta-analysis identifying risk factors for mild cognitive impairment in type 2 diabetes, highlighting the neurological dimension of diabetic complications and the need for cognitive screening in routine diabetes care.

Hepatic complications, particularly non-alcoholic fatty liver disease progressing to advanced fibrosis, are increasingly recognized in diabetes. Yaqoob et al. investigated the prevalence of high-risk for advanced liver fibrosis using non-invasive scores among type 2 diabetes mellitus patients in the United Arab Emirates, demonstrating the utility of accessible screening tools in diverse populations.

## Lifestyle interventions and novel biomarkers

Despite the proliferation of pharmacological therapies, lifestyle interventions remain foundational to diabetes management. Bao et al. demonstrated the beneficial effects of aerobic exercise on exercise capacity and quality of life in elderly patients with diabetes through a case-control study, reinforcing the importance of physical activity across the lifespan.

The exploration of novel biomarkers continues to advance the field. Wolffenbuttel et al. established reference values and identified biological factors influencing skin autofluorescence, a non-invasive marker of advanced glycation end-products that may predict future complications.

## Conclusion and future directions

The twenty studies compiled in this Research Topic collectively represent significant progress in understanding, predicting, and managing diabetes complications. Several key themes emerge: the value of novel biomarkers and risk stratification tools, the potential of artificial intelligence and machine learning in clinical prediction, the importance of integrated care addressing multiple complications simultaneously, and the persistent gap between evidence-based guidelines and real-world implementation.

Moving forward, the field must prioritize the translation of these research findings into clinical practice, develop implementation strategies to improve adherence to guideline-recommended therapies, and continue to explore personalized medicine approaches that account for individual patient characteristics, preferences, and risk profiles. The integration of digital health technologies, wearable devices, and remote monitoring systems offers unprecedented opportunities for early detection and intervention.

Ultimately, reducing the burden of diabetes complications requires a multifaceted approach encompassing prevention, early detection, evidence-based treatment, and patient-centered care. The research presented in this Research Topic provides a roadmap for clinicians, researchers, and healthcare systems to navigate these challenges and unveil new solutions that will improve outcomes and quality of life for the millions of individuals living with diabetes worldwide.

1. IDF Diabetes Atlas 11th edition 2025: global prevalence and burden of diabetes. International Diabetes Federation. 2025. Available at: https://diabetesatlas.org.
2. Huang X, et al. Global, regional, and national burden of type 2 diabetes. Front Public Health. 2025;13:1515797.
3. An update on chronic complications of diabetes mellitus. Pol Arch Intern Med. 2024;134(5):307–317.
4. Siddiqui, K., and Maddaloni, E. (2022). Editorial: The changing panorama of diabetes outcomes: novel complications and novelties in classical complications. Frontiers in Endocrinology, 12, 816481. doi: 10.3389/fendo.2021.81648
5. Editorial: Current understanding of complications associated with diabetes mellitus. Front Clin Diabetes Healthc. 2023;4:1338656.

